# Cystic Fibrosis Newborn Screening in Portugal: PAP Value in Populations with Stringent Rules for Genetic Studies

**DOI:** 10.3390/ijns4030022

**Published:** 2018-06-29

**Authors:** Ana Marcão, Celeste Barreto, Luísa Pereira, Luísa Guedes Vaz, José Cavaco, Ana Casimiro, Miguel Félix, Teresa Reis Silva, Telma Barbosa, Cristina Freitas, Sidónia Nunes, Verónica Felício, Lurdes Lopes, Margarida Amaral, Laura Vilarinho

**Affiliations:** 1 National Institute of Health Dr Ricardo Jorge, Human Genetics Department, Newborn Screening, Metabolism and Genetic Unit, Rua Alexandre Herculano 321, 4000-055 Porto, Portugal; 2 Cystic Fibrosis Center, Department of Pediatrics, Hospital de Santa Maria (CHLN), Lisbon Academic Medical Center, Av. Professor Egas Moniz, 1649-028 Lisboa, Portugal; 3 Cystic Fibrosis Center, Department of Pediatrics, Centro Hospitalar S. João, Alameda do Professor Hernâni Monteiro, 4200-319 Porto, Portugal; 4 Cystic Fibrosis Center, Centro Hospitalar de Lisboa Central, Rua Jacinta Marto, 1169-045 Lisboa, Portugal; 5 Cystic Fibrosis Center, Department of Pediatrics, Centro Hospitalar e Universitário de Coimbra, Rua Doutor Afonso Romão 3030, 3000-609 Coimbra, Portugal; 6 Cystic Fibrosis Center, Department of Pediatrics, Centro Hospitalar do Porto, Largo da Maternidade de Júlio Dinis 4050-651, Porto, Portugal; 7 Department of Pediatrics, Funchal Central Hospital, Avenida Luís de Camões 57, 9004-514 Funchal, Portugal; 8 BioISI—Biosystems & Integrative Sciences Institute, Faculty of Sciences, University of Lisboa, Campo Grande, C8, 1749-016 Lisboa, Portugal

**Keywords:** newborn screening, cystic fibrosis, IRT, PAP

## Abstract

Newborn screening (NBS) for cystic fibrosis (CF) has been shown to be advantageous for children with CF, and has thus been included in most NBS programs using various algorithms. With this study, we intend to establish the most appropriate algorithm for CF-NBS in the Portuguese population, to determine the incidence, and to contribute to elucidating the genetic epidemiology of CF in Portugal. This was a nationwide three-year pilot study including 255,000 newborns (NB) that were also screened for congenital hypothyroidism (CH) and 24 other metabolic disorders included in the Portuguese screening program. Most samples were collected in local health centers spread all over the country, between the 3rd and 6th days of life. The algorithm tested includes immunoreactive trypsinogen (IRT) determination, pancreatitis associated protein (PAP) as a second tier, and genetic study for cases referred to specialized clinical centers. Thirty-four CF cases were confirmed positive, thus indicating an incidence of 1:7500 NB. The p.F508del mutation was found in 79% of the alleles. According to the results presented here, CF-NBS is recommended to be included in the Portuguese NBS panel with a small adjustment regarding the PAP cut-off, which we expect to contribute to the improvement of the CF-NBS performance. According to our results, this algorithm is a valuable alternative for CF-NBS in populations with stringent rules for genetic studies.

## 1. Introduction

Cystic fibrosis (CF, OMIM 219700) is a monogenic, autosomal recessive, inherited, multi-systemic disease common in the Caucasian population [1,2]. It results from mutations in the CFTR (*CFTR*; MIM#602421) gene encoding the CF transmembrane conductance regulator (CFTR) protein, a chloride (Cl^−^)/bicarbonate channel functioning at the apical surface of epithelial cells [3,4]. The absence or dysfunction of the CFTR protein affects exocrine secretion, leading to a positive sweat Cl^−^ test (SCT) (a Cl^−^ concentration in sweat ≥ 60 mEq/L). Pulmonary, pancreatic and hepatobiliary symptoms, as well as male infertility, are typical signs of CF. Despite large clinical heterogeneity, respiratory tract symptoms dominate CF pathology, with early impaired pulmonary function and lung disease being the major cause of morbidity and mortality in CF [5]. Adding to the broad phenotypic spectrum, a large genotypic variety is also observed (particularly in some regions, like Southern Europe), with genotype–phenotype correlations difficult to establish due to the involvement of multiple genetic modifiers and environmental factors [6,7].

More than 2000 CFTR coding gene sequence variations have been reported (CFTR Mutation Database), but only a small percentage of these have a demonstrated CF-causative effect (www.cftr2.org, accessed on 6 April 2018) [8,9]. Despite such high genetic heterogeneity, there is one single mutation (p.F508del), which is present in 70% of CF patients worldwide, thus being the most common CF-causing mutation [10,11]. Important clinical trials are ongoing for new drugs specifically designed to improve CFTR expression and function on a mutation-specific basis, and these will likely contribute to significantly increasing the life expectancy of patients and their quality of life [6,12,13].

CF has been included in most newborn screening (NBS) programs based on well-recognized, long-term benefits [14,15,16,17]. Starting in New Zealand and Australia, it is now included in all states of the USA and most European countries [18]. Ultimately, all NBS programs refer infants with a positive screening test to a specialized CF center for SCT and clinical evaluation so as to confirm a diagnosis of CF. It is necessary to obtain evidence of CFTR dysfunction through the identification of two CFTR gene mutations previously assigned as CF-causing, two tests showing a high Cl^−^ concentration in sweat (>60 mEq/L), distinctive transepithelial nasal potential difference (NPD) measurements, and/or assessment of CFTR (dys)function in native colonic epithelia ex vivo [19,20,21,22]. Accordingly, such programs have high variability in terms of organization and algorithms. Indeed, NBS algorithms differ by country (also often by regions within the same country), but they usually start with IRT determination, with positive results triggering second-tier tests which can be either DNA analysis or PAP determination. Some algorithms also include the request of a second sample for IRT determination, and each algorithm has its own sensitivity and specificity [17]. Each program adopts an algorithm which is adapted to the characteristics of the population being tested, and matches the health system conditions of each country [6].

Here, we describe results from a three-year pilot study for CF-NBS conducted in Portugal and including 255,000 NB, with the aim of establishing the most appropriate conditions that identify the majority, if not all, of Portuguese individuals with CF and selecting the most suitable algorithm for the implementation of CF-NBS in our country, taking into account its specific features.

## 2. Materials and Methods

### 2.1. Population in the Study

This study was submitted to the ethical committee for health from the National Institute of Health Doutor Ricardo Jorge (approval date: 28 October 2013, approval number CD2013/121304554).

This pilot study for CF-NBS started at the end of 2013 and included a total of 255,000 NB. Posters and leaflets containing information regarding CF and the importance of CF screening were provided to the sampling centers, together with the sampling cards. Information regarding this study was also available at the Portuguese NBS website (www.diagnosticoprecoce.pt, accessed on 6 April 2018) and meetings about NBS are promoted for health care professionals enrolled in NBS every six months. Samples for CF screening are the same used for metabolic and CH screenings, and are mainly collected in local health centers spread all over the country between the 3rd and 6th days of life [23]. CF-NBS is carried out on a voluntary basis, with parents deciding at the time of NBS sample collection whether they want CF screening to be included in their baby’s NBS. If the parents decide to opt-out from the CF pilot study, this is indicated on the screening card. During the three years of the present study, only four couples opted out CF-NBS.

### 2.2. CF-NBS Algorithm

**Figure 1:**
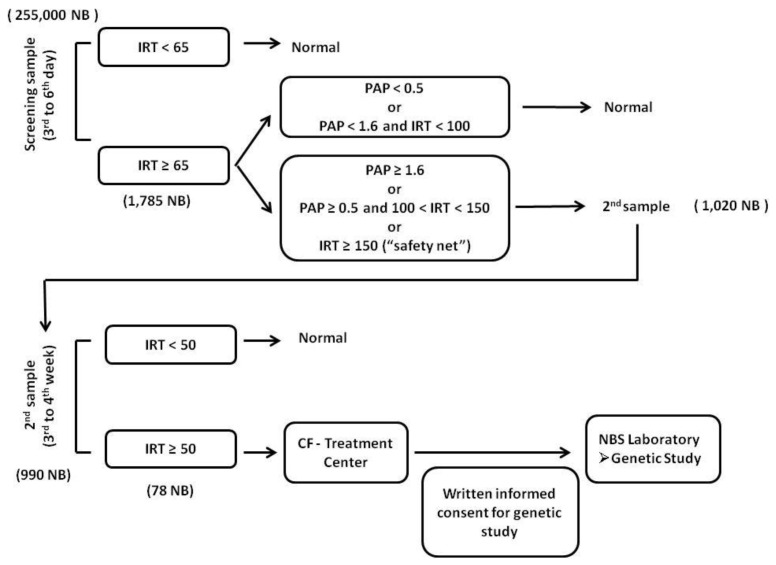
Algorithm used in the pilot study for CF-NBS in Portugal and the numbers of newborns at each stage. IRT (immunoreactive trypsinogen) and PAP (pancreatitis associated protein) are expressed in ng/mL.

In this study, an IRT/PAP/IRT algorithm was used, as shown in Figure 1. IRT quantification was performed by a sandwich time-resolved immunofluorometric assay, using the AutoDELFIA^®^ system from PerkinElmer (Waltham, MA, USA). Samples with IRT values above 65 ng/mL after repeated measurement on the same sample were selected for PAP determination. PAP quantification was also done by a sandwich time-resolved imunofluorometric assay, using the MucoPAP-F kit from Dynabio (Lyon, France). A double PAP cut-off, depending on IRT value, was used according to the manufacturer’s indication. An ultrahigh IRT cut-off (150 ng/mL) was also used for direct request of a second sample (as a “safety net” [24]), independently of PAP value.

Second dried blood spots (DBS) were typically collected in the 3rd week of life, and children presenting with IRT ≥ 50 ng/mL were referred to specialized CF clinical centers for clinical evaluation and SCT, usually at around one month of age.

Pancreatic status was determined based on the fecal elastase (FE) value and considered as pancreatic insufficient (PI) if FE < 200 µg/g, or pancreatic sufficient (PS) if higher. SCT was performed according to a standardized protocol. If the result was clearly negative (<30 mEq/L), the case was considered to be a false positive. If the result was positive (≥60 mEq/L) or borderline (≥30 and <60 mEq/L), a written informed consent for *CFTR* gene study was requested from the parents by the clinician and sent to the NBS laboratory. Genetic studies were immediately started using genomic DNA extracted from the remaining DBS blood. In a first level approach, the p.F508del mutation is tested by Amplification-Refractory Mutation System (ARMS) analysis (method adapted from Ferrie et al. [25]). If the result is not homozygosity for p.F508del mutation, genetic study proceeds using Elucigene^®^ CF-EU2v1 kit (screening for 50 Europe-frequent mutations, Elucigene Diagnostics, Manchester, UK) and Elucigene^®^ CF Iberian Panel kit (screening for 12 Iberian Peninsula-frequent mutations). In the cases of positive SCT and only one or no CF mutations identified, the complete sequencing of the *CFTR* gene by next-generation sequencing (NGS) is suggested.

## 3. Results

In this study, 255,000 NB were screened. 1785 (0.7%) were identified with an increased IRT value in the screening sample (≥65 ng/mL), but only 1020 (0.4%) met the criteria for a second sample request (Figure 1). From these, 990 samples were received (97%), mainly collected between the 3rd and 4th weeks of life. In most cases for which a second sample was not received, the newborns were very sick and premature, and were deceased. A total of 78 cases (7.9% of second samples) had persistent increased IRT results and were thus referred to a CF specialized treatment center. From these cases, 32 (41%) could be confirmed as CF. Twenty-nine cases were confirmed as being CF-positive based on a positive SCT result (Table 1).

Furthermore, three additional NB were confirmed positive for CF but not based on NBS (Table 1, patients 32–34). Patient 32 was born on Madeira Island, right after the beginning of this study and was not initially included in CF screening. At the age of six months, due to CF clinical suspicion, its screening sample was retrospectively analyzed for IRT and PAP, revealing a positive result for CF-NBS. Patient 33 was a NB who already had a positive prenatal diagnosis of CF, and who also presented with meconium ileus. In the case of Patient 34, a second sample was requested due to increased IRT and PAP, but this child was already at the hospital due to biliary atresia. Therefore, a second sample was only collected on the 49th day of life, and after several blood transfusions, which may explain the low IRT value in the second sample. Characterization of all CF-positive cases is presented in Table 1. Mutation p.F508del was present in 54/68 (79%) of the alleles; only three patients did not present with this mutation and in one case, the second mutation could not be identified (although all the CFTR exonic and flanking intronic regions had been sequenced). Complete *CFTR* gene sequencing is still in progress.

Considering the 34 cases confirmed as CF positive, a 1:7500 incidence was found for CF. This pilot study allowed the identification of an additional patient, the 7-year-old brother of Patient 1, who had a previous diagnosis of asthma. Follow-up was also recommended for a brother of an older CF-patient with a negative CF prenatal diagnosis. They presented with an elevated IRT value at screening, leading to the request of a second sample and referral to a CF center (first sample (day 6): IRT-79 ng/mL, PAP-2.0 ng/mL; second sample (day 17): IRT-65 ng/mL). Genetic study revealed a p.R334W/5T genotype, thus excluding a CF diagnosis.

From the 78 cases referred to a CF specialized treatment centre for further evaluation, 46 (59%) could not be confirmed as CF-positive (0.018% of the total screened). Of these cases, five were found to be heterozygous for CF mutations detected in the screening-included genetic study. According to the information received from CF specialized treatment centers, one positive case was missed (Patient 34). Patient 33, who had meconium ileus and a positive pre-natal diagnosis, could have been identified due to increased PAP.

During approximately four months, 30,000 NB were screened using a lower first IRT cut-off (50 ng/mL). This alteration in the algorithm led to 400 more PAP determinations and 20 additional requests of second samples. All cases presented normal IRT values in the second sample, and thus this cut-off was no longer used.

## 4. Discussion

Between 1992–1995, a pilot study for CF-NBS had already been performed for NB in the North and Center of Portugal. It included the screening of 40,000 NB, but only four CF patients were identified, corresponding to a birth prevalence of 1:10,000 NB. Due to this low value, the high number of false positive results (>1%), and to the low efficacy of the treatment available at that time, it was decided not to proceed with this screening.

In 2013, we started a second pilot CF-NBS program as a one-year collaboration project with the Portuguese National Association for Tuberculosis and Respiratory Diseases. At the end of the project, the National Technical Committee for NBS decided that the results supported the maintenance of the pilot study, with the possibility of opting out. To date, 255,000 NB have been screened for CF and during these three years, only four couples refused the participation of their children in the pilot study.

When restarting the CF-NBS pilot study in 2013, it was necessary to redefine the algorithm and cut-off values to be used to increase its positive predictive value (PPV). Several protocols with different sensitivities and specificities were reported in the literature, and to decide which one to use in Portugal, two main aspects were considered: (1) National Program’s organization, including sampling collection day, and (2) legal issues for genetic studies in our country.

The Portuguese NBS program works with no written informed consent or dissent, and although not mandatory, it has an excellent coverage rate (close to 100%). Because genetic studies cannot be done with a presumptive informed consent in Portugal, we decided not to include genetic study in the first level CF-NBS protocol. Two other factors contributed to this decision: the genetic heterogeneity predicted for the Portuguese population and the intention of minimizing the identification of healthy CF carriers and cases of CF-screening positive with an inconclusive diagnosis (CFSPID). Once the DNA tier had been excluded, we decided on an algorithm including PAP [26] because we wanted to decrease the recall rate. Compared to an IRT/IRT protocol which we had previously tested in the first pilot study in 1992, no good results were obtained due to the large number of false positive results, which affected parents’ anxiety due to prolonged uncertainty about a CF diagnosis. Although IRT quantification is widely accepted as a first marker, it is known that it is not 100% sensitive [27], and the establishment of an adequate cut-off value is a critical step in the optimization of a CF NBS program. A 65 ng/mL fixed cut-off was chosen based on the 99th percentile value calculated for 20,000 samples. Regarding the PAP cut-off value, reports available in the literature were mainly supportive of photometric determination and there wasn’t a consensual PAP cut-off value [28,29,30]. Dynabio recommendations for the fluorometric determination were adopted here. The use of PAP in our algorithm reduced the initial abnormal results from 0.7% to 0.4% (i.e., by 43%), yet a high number of cases would be considered positive. To overcome this problem, a second sample for IRT determination was requested from these cases. This procedure drastically reduced the number of false positive cases referred to clinical centers for evaluation (by 92%). Only 78 cases had IRT levels that remained elevated in the second sample (of which 46 could not thus far be confirmed CF-positive), representing a 0.018% rate of possible false positive cases referred to CF clinical centers. Performance indicators achieved for the IRT/PAP/IRT algorithm are indicated in Table 2.

PAP cut-offs adopted in this study were revealed to be too low, still resulting in a high recall rate. To make PAP a valid tier in this algorithm, PAP cut-offs should be changed. The IRT-dependent PAP cut-off also does not seem to present any advantage. No CF patient was identified with a PAP value lower than 1.6 ng/mL, and using this value as a single PAP cut-off, 161 requests of a second sample could be avoided (six of which were false positive cases referred to CF clinical centers).

The use of a safety net also contributed to the significant number of second sample requests, and all patients would have been identified without it. Based on these observations, the algorithm currently in use does not include the safety net and uses a unique 1.6 ng/mL cut-off for PAP. These changes should be revised in a period of 2–3 years, depending on the results obtained or reported by other countries.

PAP may also be a valuable tool to identify NB with meconium ileus due to CF, which are known to potentially have normal IRT values. All patients in this study presenting with this condition were found to have elevated PAP results, even in the presence of normal IRT results, which is the case in Patient 33. PAP determination is done in all cases referred due to meconium ileus, even in the presence of a normal IRT value. Identifying a large number of cases will be necessary to draw final conclusions. Any case with a suspicion of CF reported to the NBS laboratory (including NB with respiratory symptoms or meconium ileus, but with first IRT under 65 ng/mL cut-off), was retrospectively subjected to IRT re-determination, PAP determination and genetic study if written informed consent was obtained from the parents. More than 20 of these cases have been analyzed, but none were confirmed as CF positive. Currently, PAP is also determined in the second sample. It is known that it changes significantly during the postnatal period (increasing with age), but it seems to increase much more rapidly in CF patients according to some authors [31]. Although our data are too preliminary to draw a definitive conclusion, PAP values in the second sample may also be important to discriminate true positives from false positive cases.

Genetic confirmation can be a prompt and definitive way to confirm CF diagnosis, especially in cases with inconclusive SCT results. Therefore, a basic genetic study was included in the NBS protocol after written informed consent was requested by CF center clinicians from the parents. Early molecular characterization of individuals with a CF diagnosis can be vital for improved CF-specific care, with personalized treatment (mutation-targeted CFTR modulator therapy), clarification of atypical cases, and genetic counselling of risk couples regarding recurrence possibilities and prenatal diagnosis. Using this strategy, we genetically confirmed the diagnosis of 29 out of 32 screening-identified patients. Five carriers of one CFTR mutation, who could not be confirmed as CF-patients, were also found.

Regarding the genetic characterization of the CF patients, the p.F508del mutation was found to have a frequency of 79%, which is higher than expected from existing data which indicated frequencies between 52% and 63% [32,33,34]. One possibility for this is that the former values included adult non-classic CF patients that may escape NBS identification, and among which p.F508del mutations have a lower frequency. Furthermore, some severe cases (among which p.F508del has a high frequency) may not have been accounted for in the past, because they could have died before diagnosis. The first possibility is more plausible since other mutations previously reported to occur in Portuguese individuals with CF were also not represented in the current population of positive CF cases. These mutations (frequencies as in the WHO report [33]) include: A561E (3.2%); R334W (2.8%); G576A (1.4%); 711 + 1G > T and 3272–26A > G (1.0% each); Y1092X (0.8%); R74W, 621 + 1G > T, Q1100P and I1234V (0.6% each). In fact, the other non-p.F508del mutations which were identified in the current cohort of NBS positives also corresponded to frequencies slightly higher than previously described (frequencies as in the WHO Report [33]), namely: 2.9% for R1066C (2.8% before), 2.9% for G542X (2.6% before), 2.9% for G85E (2.2% before) and 4.4% for N1303K (1.8% before). Strikingly, none of the mutations detected in the current CF-NBS cohort are associated with PS, although they exist in significant frequency in Portuguese CF patients (e.g., 3272–26A > G, R334W).

CF incidence calculated from the present CF-NBS results (1:7500 NB) is lower than expected from registered patients in Portuguese CF specialized clinical centers. Similar results were found in other countries at the time of NBS implementation [17,35], which may indicate that previous estimates were not very accurate. It is also possible that some milder cases, namely PS with no early clinical manifestations, are not being identified. According to the Wilson-Jungner criteria [36], the identification of these milder late-revealing cases should not be the aim of NBS; nevertheless, their identification would be advantageous by allowing earlier action. Further investigation is needed to evaluate this hypothesis. Along these lines, we are already aware of one missed case due to blood transfusion before sampling. This case was meanwhile identified, but we know that before NBS, a significant number of CF diagnoses were done after the age of 3 years [37], so we will have to wait some more years to confirm our results and thus validate the above data.

## 5. Conclusions

According to the results presented here, CF-NBS is being included in the Portuguese NBS panel with some adjustments in the algorithm: A unique PAP cut-off will be used (1.6 ng/mL) and no safety net for ultra-high IRT will be used. With the new algorithm, we expect to improve the performance of CF-NBS screening, achieving better specificity while maintaining the sensitivity.

## Figures and Tables

**Table 1:** CF patients identified in the CF-NBS pilot study.

Sampling Day	Birth Weight (g)	Newborn Screening Results			Sweat Test (nmol/L)	Fecal Elastase (µg/g)	CFTR Gene	
		1st IRT (ng/mL)	PAP (ng/mL)	2nd IRT (ng/mL)			Allele 1	Allele 2
P1 (5, 15)	3040	94	5.4	158	116	1	p.F508del	p.F508del
P2 (21, --)	3375	130	3.9	n.d.	112	<30	p.F508del	p.F508del
P3 (6, 17)	2700	210	>8.8	287	110	<15	p.F508del	?
P4 (23, 28)	3170	190	>8.8	195	109	1	p.F508del	p.F508del
P5 (3, 13)	2555	260	4.3	344	128	<15	p.F508del	p.F508del
P6 (4, 27)	2300	266	>8.8	360	115	n.a.	p.F508del	p.F508del
P7 (5, 15)	2695	443	>8.8	531	120	<15	p.F508del	p.F508del
P8 (2, 5)	2710	101	>8.8	101	121	<30	p.F508del	p.F508del
P9 (4, 30)	2870	110	4.6	123	108	<15	p.F508del	p.F508del
P10 (3, 16)	3825	414	>8.8	265	84	<15	p.F508del	p.F508del
P11 (5, 19)	3855	238	5.2	281	93	107	p.F508del	p.R792X
P12 (4, 21)	3750	241	7.8	333	110	<15	p.F508del	p.F508del
P13 (5, 21)	2860	305	5.1	135	87	28	p.F508del	p.F508del
P14 (5, 18)	3380	156	4.7	219	95	<30	p.F508del	Ex 3 total deletion
P15 (6, 23)	3850	133	7.6	157	78	<5	p.F508del	p.F508del
P16 (3, --)	3438	91	>8.8	n.d.	90	<30	p.F508del	p.F508del
P17 (5, 27)	3320	113	5.8	133	104	<15	p.F508del	p.F508del
P18 (5, 12)	2860	463	>8.8	624	76	<5	p.F508del	p.F508del
P19 (4, 14)	1950	215	>8.8	155	100	20	p.G85E	p.R1066C
P20 (4, 12)	n.a.	255	>8.8	282	89	<10	p.F508del	p.F508del
P21 (3, 17)	3120	259	>8.8	185	n.a.	n.a.	p.N1303K	p.R1066C
P22 (3, 20)	3325	70	2.3	55	70	312	p.F508del	p.V232D
P23 (4, 23)	2560	107	7.3	191	78	25	p.F508del	p.F508del
P24 (3, 17)	2500	258	5.6	269	n.a.	<15	p.F508del	p.F508del
P25 (5, 18)	2950	240	9.3	244	88	13	p.F508del	p.F508del
P265, 18)	3265	113	≥8.8	136	83	<15	p.F508del	p.F508del
P27 (3, 15)	2760	160	9.0	172	122	72	p.F508del	p.N1303K
P28 (13, 28)	2235	67	≥8.8	51	n.a.	<5	p.F508del	p.F508del
P29 (5, 21)	3640	75	1.6	123	134	<15	p.F508del	p.R1162X
P30 (4, 15)	3100	397	≥8.8	306	69	40	p.F508del	p.G85E
P31 (3, 28)	3070	103	≥8.8	83	88	<30	p.F508del	p.F508del
P32 (4, --)	2860	202	2.7	n.d.	n.a.	n.a.	p.F508del	p.N1303K
P33 (10, 28)	3840	54	7.5	49	n.a.	<10	p.F508del	p.F508del
P34 (5, 49)	3555	108	≥8.8	11	110	<100	p.G542X	p.G542X

Legend: Patients 5 and 6 are twin brothers; P6 deceased due to cardiomyopathy; SCT was not performed for patients 28 and 33 due to the absence of adequate clinical conditions (both patients presented with meconium ileus). n.a.—not available.

**Table 2:** Performance indicators for IRT/PAP/IRT algorithm.

Screened newborns (*n*)	255,000
Confirmed CF patients (*n*)	34
False positives referred for SCT (*n*)	46
False negatives, including cases with meconium ileus (*n*)	2
Sensitivity, % [IC 95%]	94.44% [81.85–98.47%]
Specificity, % [IC 95%]	99.98% [99.98–99.99%]
PPV, % [IC 95%]	41.03% [30.78–52.11%]

## Abbreviations

ARMS	Amplification-Refractory Mutation System
CF	Cystic Fibrosis
CFTR	Cystic fibrosis transmembrane conductance regulator
CFSPID	Cystic fibrosis screen positive with an inconclusive diagnosis
DBS	dry blood spots
FE	fecal elastase
IRT	Immunoreactive trypsinogen
NB	newborn
NBS	Newborn screening
PAP	Pancreatitis associated protein
PI	pancreatic insufficient
PS	pancreatic sufficient
SCT	Sweat chloride (Cl^−^) test
PPV	Positive predictive value
